# Application of Graphite Tailings in Concrete Manufacturing: A Review

**DOI:** 10.3390/ma19030641

**Published:** 2026-02-06

**Authors:** Shan Gao, Jicheng Xu, Sijia Zhou, Man Xu, Honghao Li

**Affiliations:** 1 Shaanxi Key Laboratory of Safety and Durability of Concrete Structures, Xijing University, Xi’an 710123, China19829361030@163.com (J.X.); 2 Key Laboratory of Structures Dynamic Behavior and Control of the Ministry of Education, Harbin Institute of Technology, Harbin 150090, China; 3 School of Civil Engineering and Transportation, Northeast Forestry University, Harbin 150090, China; sijia_nefu@163.com (S.Z.); xuman306@126.com (M.X.); 4 School of Architecture and Urban Planning, Guangzhou University, 230 Wai Huan Xi Road, Guangzhou Higher Education Mega Center, Guangzhou 510006, China

**Keywords:** graphite tailings, concrete, cement mortar, mechanical property, concrete components

## Abstract

Large-scale mining of graphite, a crucial strategic mineral, generates substantial amounts of graphite tailings (GT). The stockpiling of this solid waste occupies vast land resources and poses persistent environmental risks due to potential heavy metal leaching. Repurposing GT into construction materials presents a promising solution, with its use as a partial replacement for fine aggregates in cementitious composites being one of the most effective methods. This review systematically consolidates current research on graphite tailings cement mortar (GTCM) and graphite tailings concrete (GTC). Due to its physicochemical properties comparable to natural sand, GT is suitable for producing building materials. Studies consistently demonstrate that a substitution level of 10% to 20% optimizes overall performance. This optimal range enhances particle packing, promotes cement hydration via pozzolanic activity, and refines the microstructure, leading to improved workability, superior mechanical strength, and enhanced durability, including resistance to permeability, freeze–thaw cycles, and chemical attacks. Moreover, the inherent carbon content imparts electrical conductivity to GTC, enabling functional applications like de-icing and structural health monitoring. The successful utilization of GT also extends to lightweight foamed and autoclaved aerated concrete. However, research on the structural behavior of GTC components remains limited. Preliminary findings on beams and columns are encouraging, but comprehensive studies on their seismic performance and design methodologies are urgently needed to facilitate the widespread engineering application of this sustainable material and mitigate the environmental impact of tailings accumulation.

## 1. Introduction

**Figure 1:**
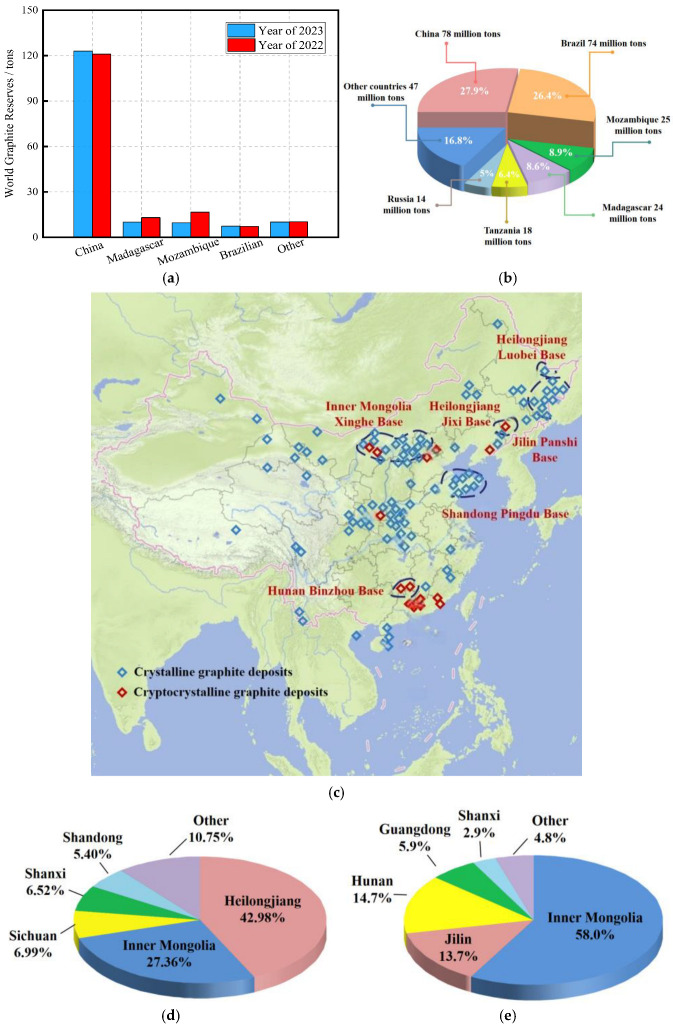

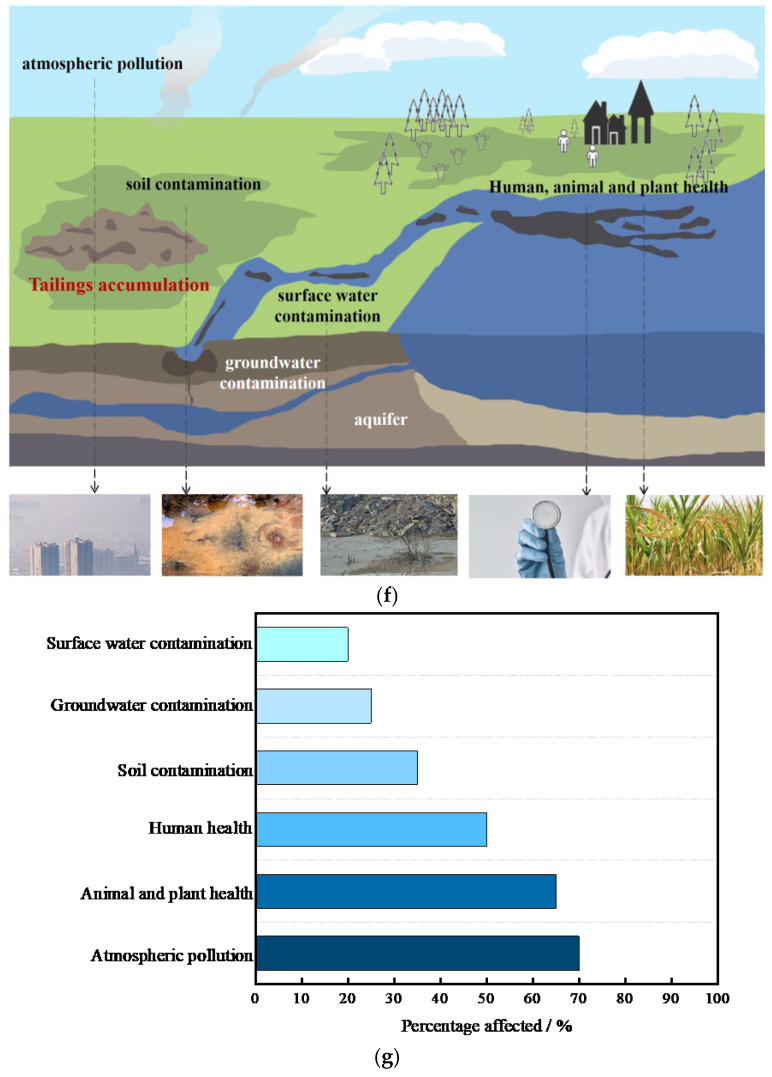
The basic situation of graphite in China and the hazards of tailings accumulation. (**a**) Global graphite production distribution [2]; (**b**) global graphite reserve distribution [2]; (**c**) distribution of graphite ore sources in China [4]; (**d**) distribution of crystalline graphite resources in China [4]; (**e**) distribution of cryptocrystalline graphite resources in China [4]; (**f**) impacts from tailings accumulation; (**g**) incidence of different impacts due to tailings accumulation [6].

Graphite, a naturally occurring elemental mineral primarily composed of carbon, possesses a unique structure that imparts exceptional physicochemical properties, including high-temperature resistance, corrosion resistance, excellent plasticity and ductility, as well as superior electrical and thermal conductivity [1]. Consequently, graphite is regarded as a crucial strategic resource for national defense, modern industry, and high-technology applications, earning the nickname “industrial monosodium glutamate.” The global distribution of graphite resources is highly concentrated. According to the U.S. Geological Survey (USGS), as of 2023, China holds graphite reserves of 78 million tons, accounting for 27.86% of the world’s total, ranking first globally. Brazil follows with 74 million tons (26.43%) in second place [2]. In 2023, China, Madagascar, and Mozambique were the three largest graphite producers, collectively contributing nearly 89% of global output, with annual productions of 1.23 million tons, 100,000 tons, and 96,000 tons, respectively [2]. The global graphite production and reserve distributions are illustrated in Figure 1a,b. Figure 1a,b illustrates global graphite production and reserve distributions. The graphite industry has become a foundational sector for global industrial development; as of 2018, China led the world in both natural graphite production and exports, while ranking second in imports [3]. Graphite is classified into crystalline and cryptocrystalline forms, with crystalline graphite notable for its high electrical and thermal conductivity and robust mechanical strength, making it highly versatile. Within China, graphite resources are widely distributed, with major concentrations in Heilongjiang and Inner Mongolia, as shown in Figure 1c–e [4].

The large-scale extraction of graphite produces substantial amounts of industrial waste, particularly graphite tailings (GT), which are solid residues generated during the beneficiation process. As the world’s largest graphite producer, China generates over 6 million tons of GT annually [5]. Despite their abundance, these tailings are largely underutilized. Studies indicate that the chemical elements in GT gradually leach out over time, resulting not only in the occupation of extensive land around graphite processing plants but also in severe contamination of water bodies and the atmosphere, thereby posing risks to human, animal, and plant health. As shown in Figure 1f,g, the massive accumulation of GT has become a critical issue restricting regional economic development and threatening human well-being [6,7]. Consequently, developing effective treatment and utilization methods for GT has emerged as a key research focus for scholars.

GT have been extensively investigated and employed as a partial substitute for concrete aggregate. However, research on the application of graphite tailings concrete (GTC) in structural components such as beams, columns, and walls, as well as the corresponding design methodologies, remains scarce and fragmented. Despite growing interest in GT utilization over the past decade, no comprehensive review has synthesized the microstructural, mechanical, durability, and structural findings into a coherent framework to guide future research and engineering practice. This review therefore aims to: (1) systematically consolidate current knowledge on GTCM and GTC; (2) critically evaluate the underlying mechanisms and performance limitations; (3) highlight gaps in structural application and durability modeling; and (4) propose targeted research directions to accelerate the sustainable utilization of GT in construction.

This review is based on a comprehensive literature survey conducted using major academic databases, including Web of Science, Google Scholar, and CNKI. The literature search employed keywords such as ‘graphite tailings’, ‘graphite tailings concrete’, ‘graphite tailings cement mortar’, and ‘solid waste utilization in cement-based materials’. Peer-reviewed journal articles were prioritized, with emphasis on studies addressing physical properties, mechanical performance, durability, and engineering applications of graphite tailings in cement mortar and concrete. Representative earlier studies were also included to ensure completeness.

## 2. Utilization of Graphite Tailings

For a prolonged period, GT have been managed through relatively limited approaches. In China, the primary methods involve stockpiling or direct utilization as roadbed landfill material [8], which, although simple and convenient, do not effectively promote resource reuse and may pose environmental risks. With technological advancements, researchers have exploited the characteristics of GT and graphite, as well as the recovery of other valuable minerals within the tailings, to transform stockpiled GT into reusable and clean resources. Techniques employed include secondary recycling, secondary utilization, and the production of ceramics and construction materials.

**Figure 2:**
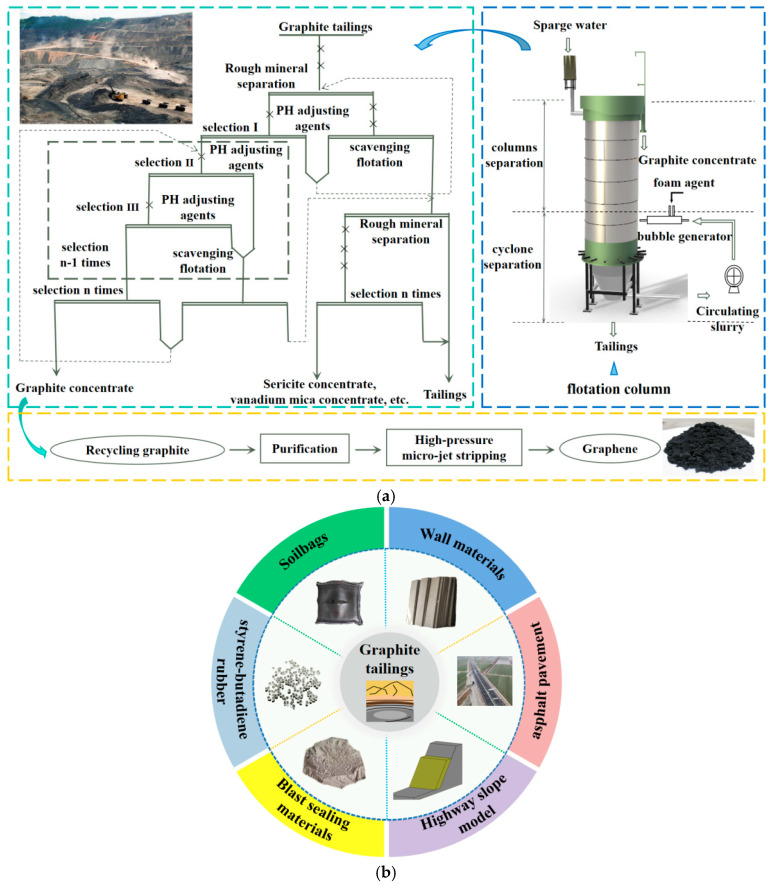
Example of GT application. (**a**) Recycling of GT using flotation methods; (**b**) examples of other applications of GT.

Given the abundant content of various minerals and elements in GT, their resource utilization is of great significance for sustainable development. As illustrated in Figure 2a, graphite concentrate, sericite, and vanadium mica concentrate can be extracted from GT via flotation methods. High-quality graphene materials can be prepared from recovered graphite. A comprehensive review indicates ongoing challenges in high-end innovation and scale-up, alongside expanding potential in construction [9]. Graphite-derived materials enhance mechanical performance, durability, and functional properties like conductivity in cementitious systems [10], with similar benefits reported for GT composites [11]. Efficient beneficiation enables graphite recovery from GT for high-value use [12], while mineral separation and modification improve overall recycling feasibility [13]. Complementary recovery of sericite and other components supports multi-resource utilization of GT [14,15].

Using GT as the main raw material, along with clay, coal slurry, kaolin, quartz, feldspar, etc., various ceramic building products can be produced. Studies show GT-based ceramic bricks achieve high flexural strength and low water absorption under suitable sintering conditions [16], with properties strongly dependent on sintering temperature [17]. Building ceramics primarily made from GT meet relevant national standards [18], and feasibility has been systematically verified [19]. Recent work further demonstrates that GT can be used to produce foamed ceramics via self-foaming [20], unburned imitation stone bricks [21], and sintered bricks with controlled efflorescence [22]. Beyond ceramics, dry stacking has been proposed to reduce ecological risks and improve water recycling [23], and treated GT can partially replace sand in concrete without compromising strength or durability [24].

Figure 2b illustrates additional engineering applications of GT. The load-bearing feasibility of GT-filled earth bags has been experimentally shown [25], with compression behavior and capacity influenced by fill density, bag dimensions, and tensile strength [26,27]. In polymer modification, GT-derived composite powders enhance tensile strength and conductivity of styrene–butadiene rubber [28]. For pavement engineering, GT functions effectively as filler in asphalt mastics [29] and, combined with carbon fibers, enables conductive asphalt mixtures with improved electrothermal performance [30]. Cement-stabilized GT exhibits satisfactory compressive strength for roadbeds [31] and stable performance under freeze–thaw cycles [32]. The production of pavement bricks using GT as a partial aggregate replacement has also been demonstrated [33]. Overall, GT use in pavement engineering supports environmental protection and sustainable infrastructure development. Additionally, GT can be utilized in the preparation of blasting sealing materials with superior performance and as backfill material for ecological restoration of road slopes [34,35]. After evaluating the advantages and limitations of GT in wall material production, it has been concluded that with technical advancements, their use in this application holds a certain degree of feasibility [36].

Beyond the aforementioned recycling techniques, one of the most effective approaches involves the incorporation of GT into construction materials. The utilization of GT has facilitated the development of graphite tailings cement mortar (GTCM) and graphite tailings concrete (GTC) with enhanced performance [37]. Owing to the compositional similarity between GT and natural river sand, substituting GT for natural sand as a concrete aggregate offers an environmentally sustainable solution. Furthermore, GT can be applied to concrete structures, significantly enhancing the performance of modified concrete. Compared to ordinary concrete, the incorporation of an appropriate amount of GT can improve durability-related properties such as permeability resistance, chloride ion penetration resistance, and sulfate attack resistance. These improvements are closely associated with microstructural densification and pore refinement, which are generally regarded as beneficial for extending the service life of concrete components [38,39,40,41].

## 3. Properties of Graphite Tailings

### 3.1. Physical Properties

**Figure 3:**
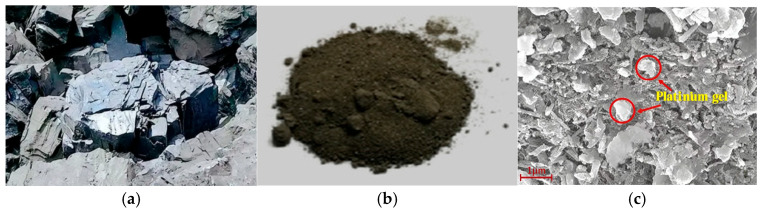
Characteristics of GT. (**a**) Graphite ore; (**b**) graphite tailings [10]; (**c**) microscopic characteristics of GT [20].

Graphite, an allotrope of carbon, possesses notable physicochemical properties, including corrosion resistance and high thermal and electrical conductivity. The mining of graphite ore generates substantial quantities of GT. As shown in Figure 3, natural graphite ore is black, exhibiting a scaly metamorphic and lamellar structure, primarily composed of graphite flakes with a semi-metallic luster. GT generally appears as gray-black or gray-green fine-grained material, with particle sizes dependent on the beneficiation process. Microscopic observations indicate that the GT surface is porous, rough, and exhibits uneven particle size distribution, where the arrangement of graphite crystals significantly influences the material properties of GT [42].

The particle size distribution of GT from the Jixi and Luobei regions was analyzed, as summarized in Table 1 [43]. The coefficient of uniformity (Cu) of GT in the Jixi area is 5.60, and the coefficient of curvature (Cc) is 1.21; for Luobei, the Cu is 5.36, and Cc is 1.23. Both types of GT exhibit well-graded characteristics, with Cu > 5 and Cc between 1 and 3. The fineness modulus of GT from different regions is similar, approximating 0.9 [44]. According to the classification of construction sand [45] in GB/T 14684-2011, GT can be categorized as superfine sand. The main physical properties of GT from the selected regions are summarized in Table 2 [46].

**Figure 4:**
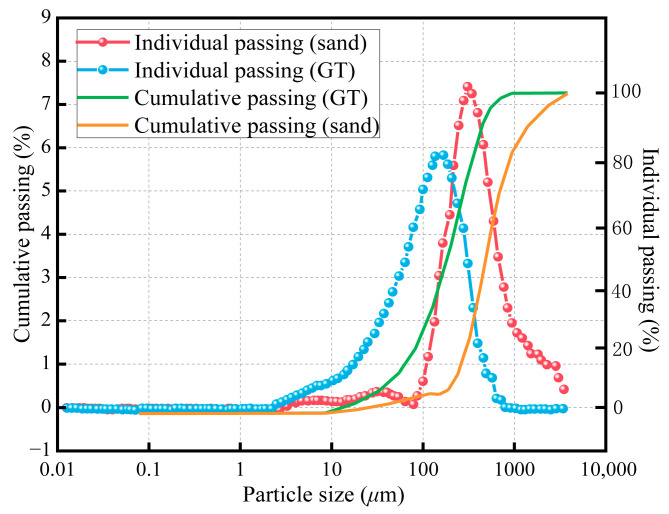
Comparison of the particle size distribution (sieve curves) of typical GT and standard natural sand.

The particle size distribution, or sieve curve, of the materials is a key factor influencing the packing density and water demand of cementitious mixtures. As illustrated in Figure 4, GT typically exhibit a finer grading compared to standard natural sand. While the specific distribution depends on the source and processing method, the majority of GT particles are concentrated in the finer range (0.075–0.6 mm), often resulting in a lower fineness modulus. This finer characteristic facilitates a micro-filler effect, which can densify the interfacial transition zone, although it may simultaneously increase the water requirement of the mixture due to the increased specific surface area.

### 3.2. Chemical Properties

Table 3 summarizes the mineralogical composition of GT, whereas Table 4 presents their chemical composition. Analysis of the mineral composition reveals that GT primarily consist of minerals such as graphite, mica, quartz, and feldspar. The main chemical constituents in GT include silicates, alumina, iron-containing minerals, calcium-containing minerals, and magnesium-containing minerals, among others [51].

### 3.3. Heavy Metal Pollution

After prolonged open-air storage, toxic heavy metals in GT can leach into the surrounding soil via atmospheric deposition and water infiltration, potentially spreading to adjacent areas and causing heavy metal contamination. Therefore, it is essential to monitor soil pollution levels and implement appropriate mitigation measures. Soil heavy metal pollution can be described using the pollution index (*P_i_*), which can be calculated as follows:

(1) $$P_{i}=C_{i}/S_{i}$$

where *P_i_* is the pollution index of pollutant *i* in soil; *C_i_* is the measured concentration of pollutant *i* (mg/kg); and *S_i_* is the background or control site value or standard value of pollutant *i* in soil (mg/kg). The classification criteria are shown in Table 5.

Unless otherwise stated, discussions of heavy-metal content and environmental risk refer to untreated GT. Studies reporting reduced radioactivity or leaching behavior are based on GT subjected to thermal treatment or encapsulation within cementitious matrices, which significantly alter their environmental performance.

Several representative heavy metal elements, including Zn, Ni, Cr, and Hg, were selected to assess the pollution levels in GT. Table 6 presents the measured concentrations and the corresponding single-factor pollution indices of these elements [53]. The results indicate that, based on total heavy metal content, the pollution degree follows the order Zn > Cr > Ni > Hg. However, when evaluated using the single-factor pollution index, the ranking changes to Hg > Ni > Zn > Cr, with Ni classified as moderately polluted, Zn as lightly polluted, Cr as unpolluted, and Hg as heavily polluted. Therefore, Hg is identified as the primary heavy metal pollutant in GT. Accordingly, when employing GT as a partial replacement in cement mortar or concrete, it is crucial to verify that the heavy metal concentrations comply with relevant regulatory standards. Pre-use monitoring and assessment are recommended to ensure no adverse effects on the ecological environment or human health.

Standard leaching test methods, such as TCLP or similar national protocols, should be employed to evaluate whether the leachate concentrations comply with relevant regulatory limits. The encapsulation of GT within the cementitious matrix can significantly reduce heavy metal mobility due to chemical fixation and physical encapsulation. However, for applications involving high-volume utilization or exposure to aggressive environments (e.g., acid rain), pre-treatment of GT (such as thermal activation or washing) or the use of immobilizing admixtures may be recommended. Pre-use monitoring and a risk assessment based on leaching behavior, rather than total content alone, are essential to ensure no adverse impacts on the ecological environment or human health.

### 3.4. Comparison with Natural River Sand

**Figure 5:**
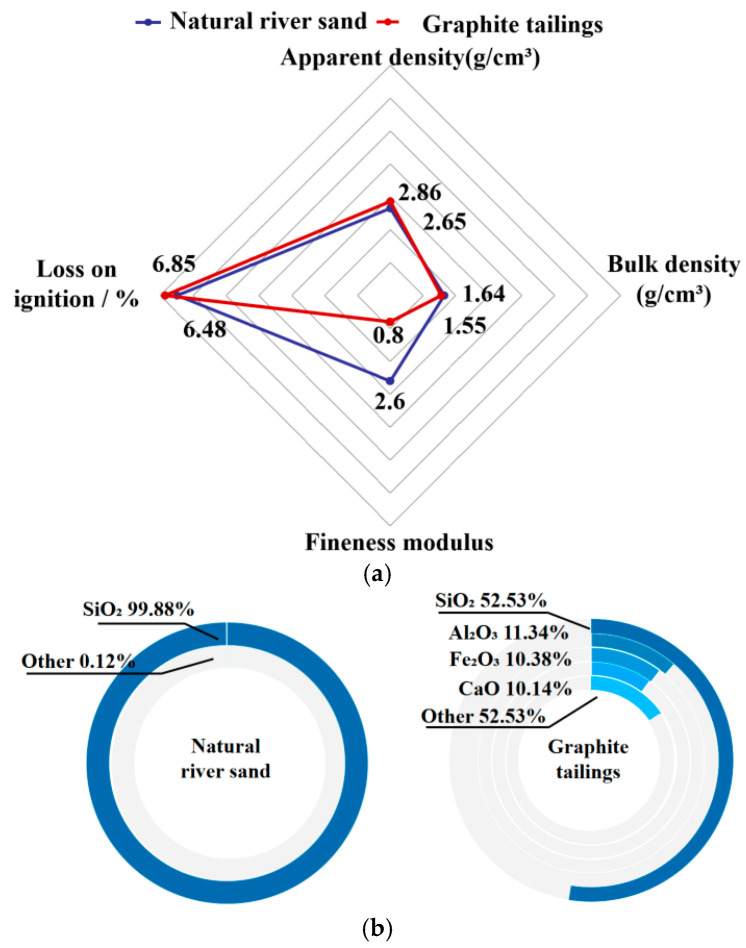
Comparison of physical properties and chemical composition of natural river sand and GT. (**a**) Physical properties; (**b**) chemical composition.

Figure 5 compares the physical and chemical properties of GT and ordinary river sand. It is evident that the physical properties of GT are comparable to those of natural river sand, satisfying the relevant standards for construction sand [42]. Regarding chemical composition, both materials primarily consist of silicates, indicating substantial similarity. Moreover, Tang et al. (2022) [38] reported that the radioactivity of GT markedly decreases after calcination at 750 °C, suggesting that building materials prepared with GT pose no significant environmental or biological hazards. Therefore, it is feasible to utilize GT as a partial replacement for natural river sand in the preparation of cement mortar and concrete.

### 3.5. Discussions

Reported physical and chemical properties of GT vary significantly due to differences in ore sources and beneficiation processes, which limits direct comparison across studies. Most studies consistently show that GT particles are finer than natural sand, supporting a micro-filling and particle packing effect in cementitious systems, which can densify the matrix and improve interfacial characteristics at low replacement levels. However, chemical composition alone is insufficient to infer pozzolanic reactivity, and existing studies provide limited direct evidence of chemical contribution. Although SEM observations frequently indicate a denser microstructure and reduced pore connectivity in GT-modified systems, these features should primarily be interpreted as the result of physical densification rather than strong intrinsic reactivity, a conclusion further supported by reported reductions in porosity and permeability. In contrast, mineralogical evidence from XRD remains scarce and often inconclusive, as GT phases largely overlap with inert siliceous and aluminosilicate minerals, making it difficult to directly identify hydration products attributable to GT. Therefore, improvements in mechanical and durability performance reported in the literature should be mainly attributed to particle packing, micro-filling, and hydration acceleration effects rather than the formation of new crystalline phases, highlighting the necessity of clearly distinguishing physical effects from intrinsic chemical reactivity when interpreting GT performance.

## 4. Properties of Cement Mortar Containing Graphite Tailings

**Figure 6:**
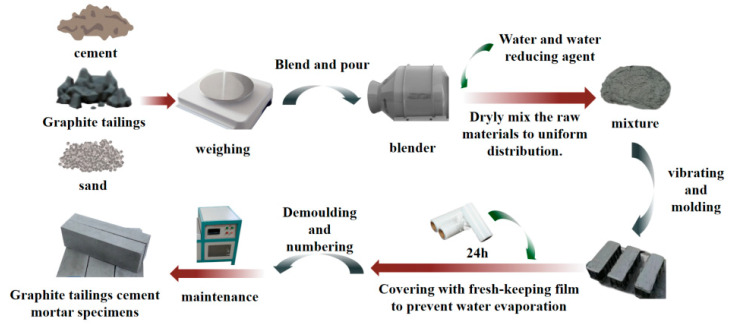
Preparation process of GTCM specimen.

GT, as a novel type of non-metallic solid waste material exhibiting certain pozzolanic activity, can promote the formation of conformationally stable and more abundant hydration products, thereby mitigating the development of initial imperfections in cement-based composites [57]. Incorporating GT as a partial replacement for natural river sand in cement mortar is not only technically feasible but also offers an environmentally friendly and cost-effective approach for GT valorization [58]. The preparation process of cement mortar specimens using GT is illustrated in Figure 6.

### 4.1. Water Absorption and Porosity

Since water molecules can easily penetrate into the interior of building materials, it is crucial to study the hygroscopic property, impermeability, and frost resistance of hygroscopicity, impermeability, and frost resistance of materials to evaluate their service life and enhance mechanical performance. Graphite tailings exhibit high water retention capacity, and the finer GT particles interact with larger cement grains at the paste–aggregate interface, promoting a dense packing of solid components in the cement mortar. This reduces porosity and improves the overall quality and durability of the material.

**Figure 7:**
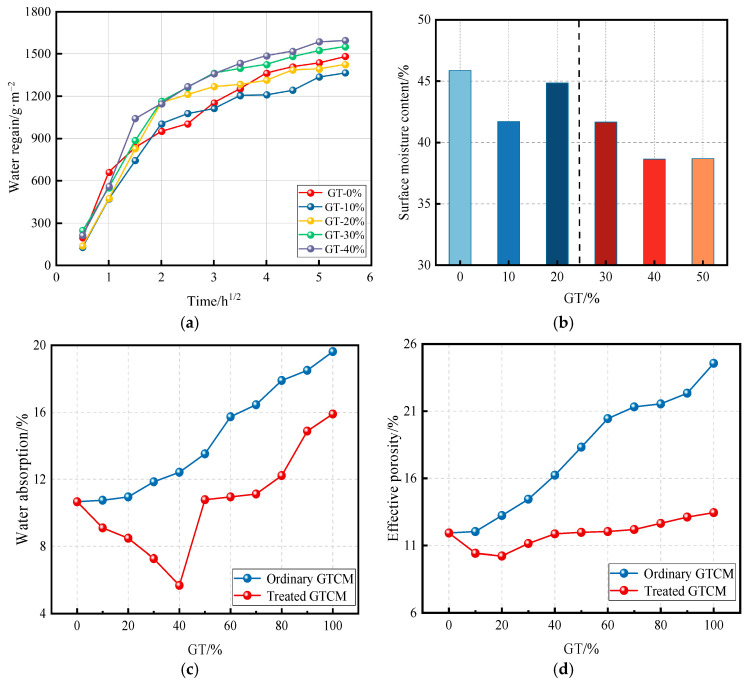
Working performance of GTCM. (**a**) Water absorption [59]; (**b**) surface moisture content [60]; (**c**) water absorption of two kinds of GTCM [22]; (**d**) effective porosity of two kinds of GTCM [22].

As shown in Figure 7a, the water absorption of cement mortar reaches a minimum at 10% and 20% GT incorporation, indicating that water diffusion in the mortar is lowest at these incorporation levels. This results in a denser surface structure and, consequently, superior impermeability performance [59]. Figure 7b demonstrates the effect of different graphite tailings’ incorporation on the surface moisture content of cement mortar. When GT content is below 40%, the surface moisture content is relatively higher, as the ultrafine GT particles reduce the connectivity of larger pore spaces, thereby lowering the overall water absorption rate. However, at GT incorporations exceeding 40%, the tailings interact with a large amount of free water, causing an uneven pore distribution, increased pore water pressure, and, ultimately, a rise in water absorption rate along with a decrease in surface moisture content. Therefore, excessive GT incorporation (>40%) leads to a decline in surface moisture content of the GTCM [60].

The graphite content can be removed by heating the GT in a muffle furnace at 1000 °C [22]. Figure 7c,d presents the water absorption and effective porosity of cement mortar prepared with untreated GT and high-temperature-treated GT, respectively. The treated GTCM exhibits lower water absorption and reduced effective porosity across all substitution levels (10–100%). This phenomenon arises because unburned graphite absorbs water, leading to higher porosity and water absorption. In contrast, the combustion of graphite produces finer particles, which fill the pores within the mortar, thereby decreasing both porosity and water absorption.

In general, as the proportion of graphite tailings increases, the impermeability of cement mortar initially improves and subsequently declines. At a GT replacement rate of 20%, the cement mortar exhibits optimal impermeability, characterized by a well-distributed pore structure and minimal porosity. Therefore, when utilizing GT as a partial replacement for natural river sand and gravel in cement mortar, it is crucial to select an appropriate substitution level to achieve enhanced performance.

### 4.2. Mechanical Performance

**Figure 8:**
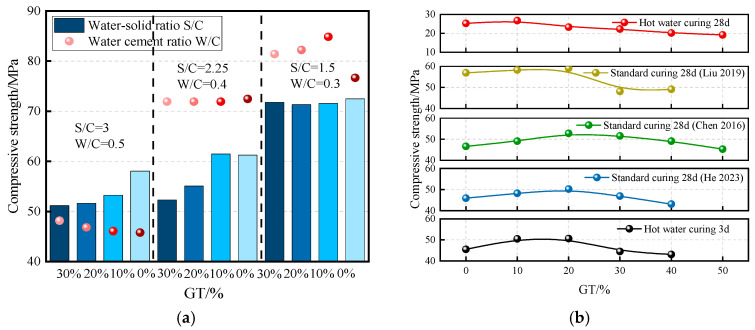

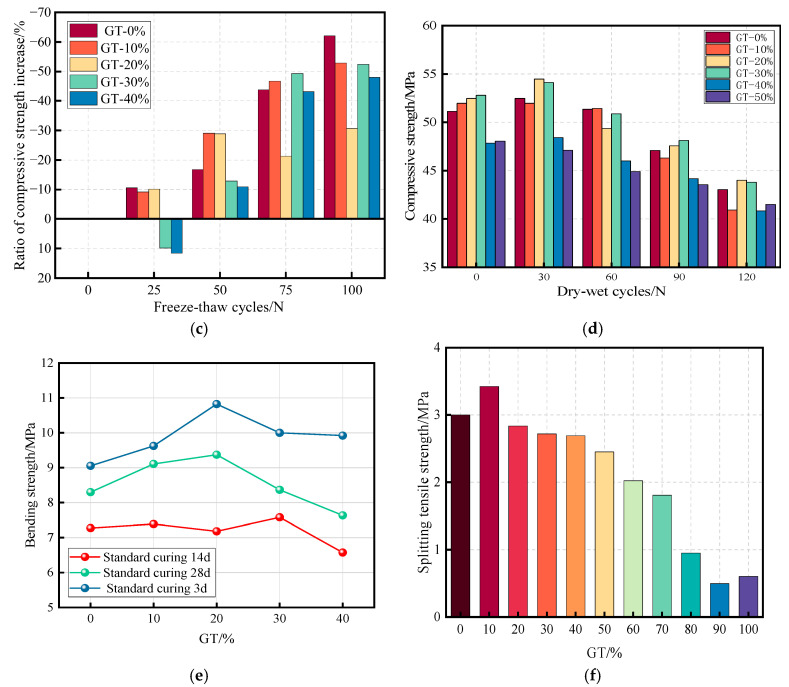
Mechanical properties of GTCM. (**a**) Compressive strength under different mix proportions [61]; (**b**) compressive strength under different curing methods [61], Data sources (from top to bottom): [22,39,59,60,64]; (**c**) compressive strength under different freeze–thaw cycles [40]; (**d**) effect of sulfate attack on compressive strength [62]; (**e**) bending strength [60], Data sources (from top to bottom): [39,60,64]; (**f**) splitting tensile strength [22].

Liu et al. (2022) [61] investigated the variation law of strength, porosity, and pore size distribution of GTCM. As shown in Figure 8a, at low water–cement ratios and water–solid ratios, the incorporation of GT influences the mechanical properties of cement mortar. Numerous studies have demonstrated that the inclusion of a moderate amount of GT can enhance the compressive strength of cement mortar. Figure 8b summarizes the effects of different GT replacement rates on the compressive properties of cement mortar reported by various scholars. The compressive strength of GTCM with 20% GT was reported as 50.1 ± 1.8 MPa (mean ± SD, *n* = 5) [59]. Overall, the compressive strengths of GTCM subjected to standard curing and hot water curing exhibit similar trends, with optimal compressive performance observed at a GT content of approximately 20%.

The effect of GT incorporation on cement mortar under different freeze–thaw cycles is shown in Figure 8c. Cement mortars containing 30% and 40% GT exhibited satisfactory performance at 25 freeze–thaw cycles. However, as the number of cycles increased, cement mortar with a 20% GT incorporation demonstrated a noticeable advantage. Higher GT content leads to the formation of additional pores within the material, whereas at 20% incorporation, the internal composition of the material is well distributed without excessive defects, yielding optimal performance [40]. Figure 8d shows the compressive properties of GTCM under dry–wet cycles of sulfate erosion. By studying the mechanism of change, it was revealed that with continuous sulfate erosion, a large amount of crystallization occurs within the material, leading to cracks within the matrix. While basalt fibers possess high tensile strength, their appropriate incorporation into cement mortar can counteract the expansion stresses and inhibit crack propagation [62]. The optimal compressive performance of cement mortar specimens was observed with 20% GT and 0.3% basalt fiber.

As shown in Figure 8e, the effect of GT incorporation on the flexural strength of GTCM has been investigated by several scholars [60]. The results indicate that the flexural strength initially increases and subsequently decreases with increasing GT substitution, reaching a maximum at a replacement rate of 20%. Excessive replacement of natural river sand with GT leads to a reduction in mechanical performance. This behavior can be attributed to the strong water absorption capacity of GT in the dry state, which reduces the effective water–cement ratio and enhances early-stage strength. However, as the GT replacement rate further increases, the water-reducing admixture becomes overly absorbed, and the accumulation of sulfide compounds in GT adversely affects the mechanical properties of GTCM.

GT had a significant effect on the bending strength of each curing method. However, the standard curing method has an inhibitory effect on the strength of GTCM compared to the hot water curing method. It can be found that the strength of the standard curing method tends to decrease more significantly with increasing GT content. This is due to the fact that the C-S-H generated during the standard curing process hinders the diffusion of cement particles such as tricalcium silicate in solution [63], resulting in weaker internal stresses in the mortar, which leads to a decrease in bending strength.

The influence of GT incorporation on the splitting tensile strength of GTCM was investigated by Kathirvel et al. (2018) [22]. As shown in Figure 8f, the splitting tensile strength of mortar with 10% GT exhibited a slight improvement. However, further increases in GT content led to a reduction in splitting tensile strength. Therefore, a 10% GT incorporation provides the optimal splitting resistance for GTCM.

In general, when the GT incorporation is approximately 20%, the various properties of GTCM can reach the best state. However, research on GT to change the performance of cement mortar is still in its infancy, and further investigation is needed on how GT affect the early strength of cement-based materials and related maintenance methods.

### 4.3. Durability

This section reviews the durability aspects of GTCM, focusing on freeze–thaw resistance, drying shrinkage, and high-temperature resistance, as these properties have been the most extensively investigated in the existing literature.

**Figure 9:**
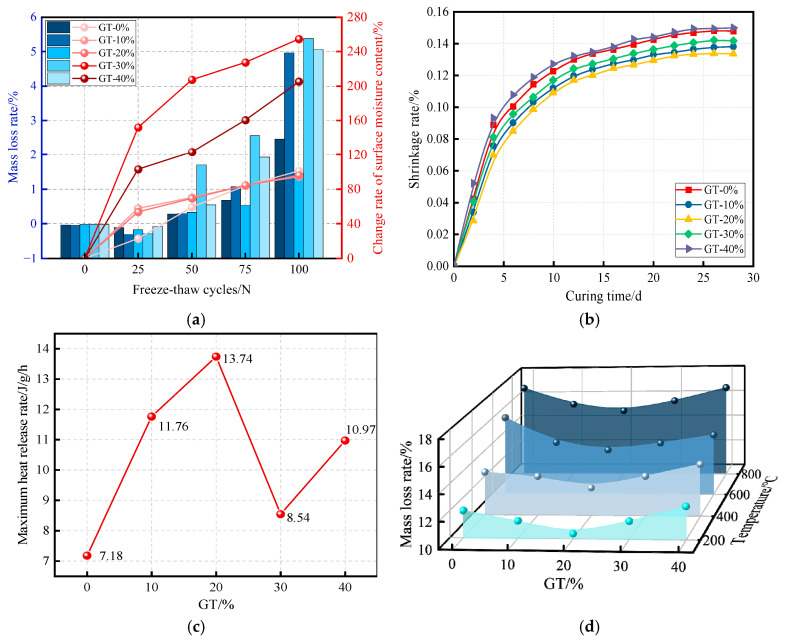
Evaluation of GTCM durability: mass loss, shrinkage, and thermal response. (**a**) Mass loss and change rate of surface moisture content under different freeze–thaw cycles [40]; (**b**) shrinkage rate under different curing times [64]; (**c**) maximum heat release rate under high temperature [57]; (**d**) mass loss rate under high temperature [57].

Figure 9a presents the mass loss rate of cement mortar and the rate of change in surface moisture content for varying GT incorporation levels under different freeze–thaw cycles. According to relevant specifications and empirical experience, a mass loss rate exceeding 5% indicates a loss of frost resistance in cement-based materials, emphasizing the need to select an appropriate GT replacement ratio. When GT are incorporated in suitable amounts, they promote better agglomeration of cement and water, enhancing the hydration process and thereby improving the early-age strength of the mortar. Consequently, the incorporation of GT can influence and potentially enhance the frost resistance of cement mortar [40].

However, GT have certain limitations in improving the toughness of cement-based materials. As depicted in Figure 9b, a moderate amount of GT blending can bolster the early strength of cement mortar and improve its shrinkage resistance, thereby impeding matrix deformation and retarding cement mortar shrinkage. Conversely, excessive GT doping can diminish the early strength of cement mortar [64]. The best drying shrinkage performance was observed with 20% graphite tailings incorporation. Yuan et al. (2024) [65] discovered that the addition of an appropriate amount of basalt fibers to GTCM could prevent sulfate ion infiltration, refine the pore structure, and substantially mitigate durability degradation in cement mortar. This finding holds great promise for broadening the application scope and practical engineering use of GTCM.

The exothermic rate during clinkerization serves as an indicator of cement mortar activity. The low water permeability of GT increases the effective surface area of solid particles in the cement mortar, thereby accelerating the cement hydration reaction. A specimen with 20% GT incorporation achieves a more favorable balance in the timing of the exothermic heat release rate peak, promoting enhanced cement mortar hydration. This, in turn, provides a favorable material configuration and foundation for withstanding high-temperature complex environments, as illustrated in Figure 9c. The mass loss rate provides insight into the extent of high-temperature damage to the cement mortar interiors. Figure 9d illustrates the relationship between different GT incorporation and mass loss under varying high-temperature conditions. Below 200 °C, free water evaporation leads to rapid mass loss in cement mortar; as the temperature increases, the decomposition and transformation of hydration products become crucial contributors to mass loss. Cement mortar with a 20% GT incorporation exhibits the least damage, as an appropriate amount of GT promotes cement hydration. Conversely, excessive GT addition leads to the decomposition of hydration products, generating numerous pores due to slurry contraction. This mismatch with the deformation of continuously expanding aggregates results in micro-cracks, water migration, and a reduction in cement mortar quality.

### 4.4. Discussions

Mechanical performance of GT-incorporated cement mortar and concrete generally improves or remains comparable at low replacement levels (typically 10–20%), but deteriorates as the GT content further increases. Contrasting findings reported in the literature can be attributed to the competition between beneficial particle packing and micro-filling effects at low replacement levels and adverse dilution effects associated with the limited intrinsic reactivity of GT at higher contents. As the GT content increases, the reduction in effective cementitious material and insufficient formation of hydration products become dominant, leading to strength deterioration. In addition, variations in mix design parameters, particle size distribution, and curing conditions further contribute to discrepancies among reported results. These observations indicate that GT content alone is insufficient to predict mechanical performance without considering mixture composition and curing regimes.

## 5. Properties of Concrete Containing Graphite Tailings

With the burgeoning development of China’s construction industry, the rapid increase in buildings and infrastructure has led to a growing shortage of concrete aggregates. Concurrently, the extensive extraction of mineral resources has resulted in a massive accumulation of tailings, posing a significant environmental and social burden. Research has revealed that GT, after crushing and sieving, can be processed into alternative raw materials for concrete production, thereby reducing the overall cost of concrete and promoting the reutilization of solid waste resources. Graphite, characterized by excellent corrosion resistance and superior thermal and electrical conductivity, imparts both chemical stability and mechanical strength to GT. Additionally, GT possess high calorific value and electrical conductivity, and their incorporation into concrete can enhance its mechanical performance and durability. Utilizing GT as a supplementary raw material in concrete not only reduces the need for landfilling but also mitigates associated environmental impacts. The manufacturing process of GT-based concrete is essentially identical to that of graphite tailing cement mortar (GTCM).

### 5.1. Workability

**Figure 10:**
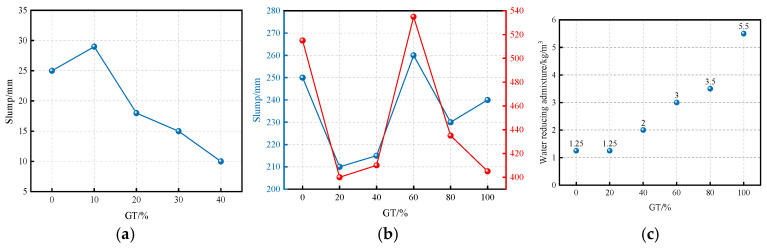
Working performance of GTC. (**a**) The slump of ordinary GTC [66]; (**b**) slump and expansion degree of concrete with high content of graphite tailings [65]; (**c**) the dosage of water reducing admixture for concrete with a large amount of graphite tailings [65].

The workability of GTC is shown in Figure 10. It demonstrates that incorporating GT reduces the concrete slump [65,66]. This reduction occurs for two main reasons. On one hand, GT particles, which are smaller and have a larger specific surface area than ordinary river sand, modify the particle interactions within the concrete. On the other hand, GT also delays the cement setting time. Both effects collectively contribute to the decrease in slump. Consequently, as the GT content increases, the dosage of the water-reducing admixture must be adjusted to maintain a balance between slump and flowability.

### 5.2. Mechanical Performance

**Figure 11:**
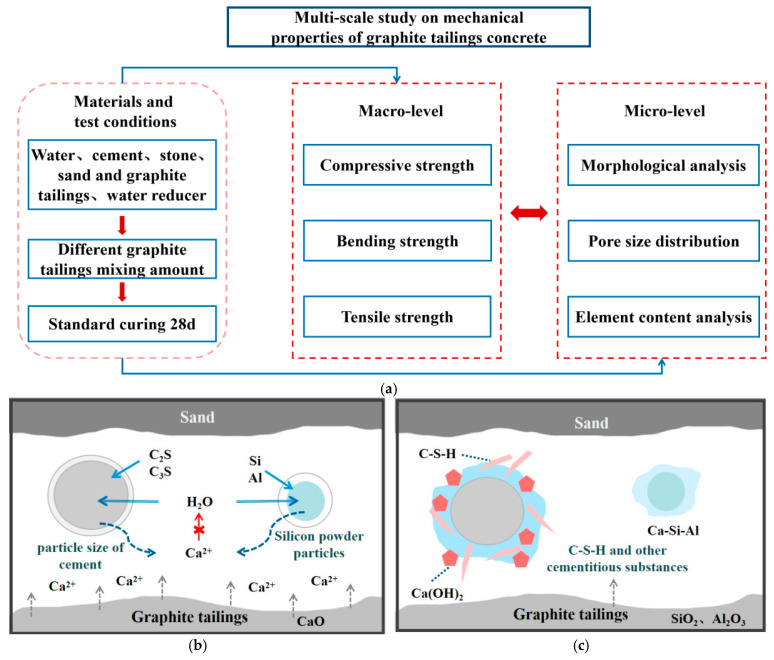

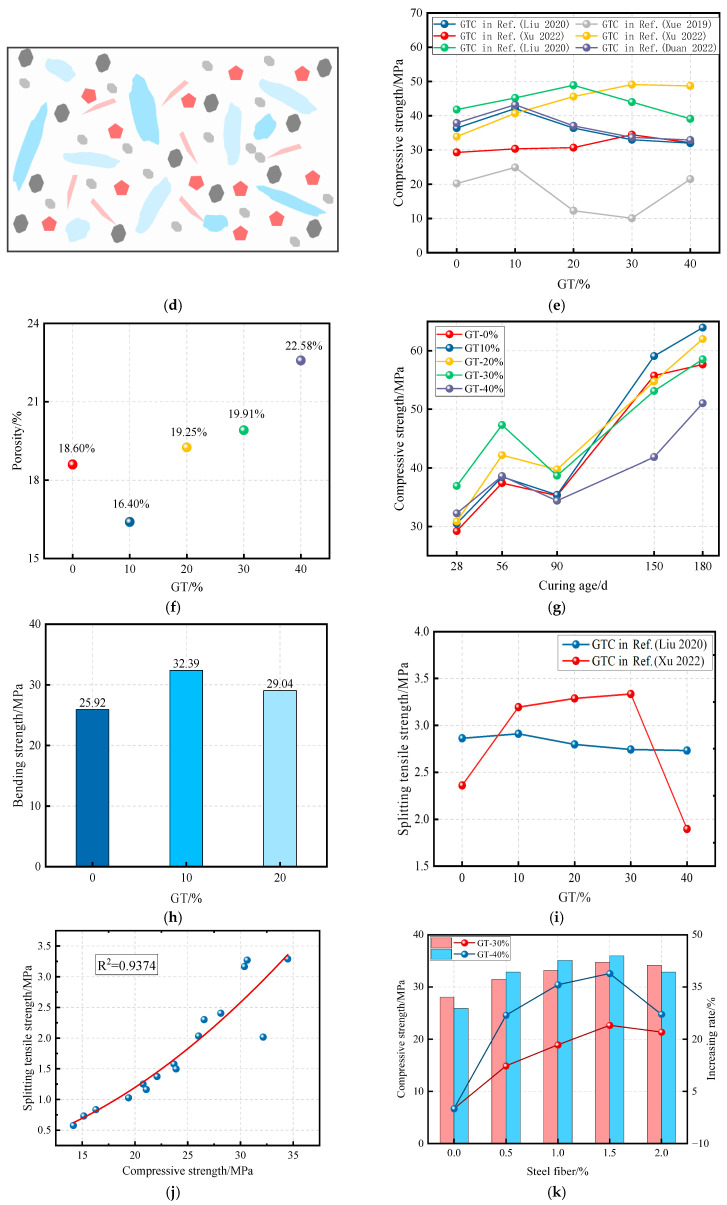
Mechanical properties of GTC. (**a**) Multi-scale study on mechanical properties of GTC; (**b**) hydration mechanism of cement-based materials; (**c**) hydration and pozzolanic reaction; (**d**) internal structure of GTC; (**e**) compressive strength of GTC [46], Data sources (from left to right and top to bottom): [46,67,68,69,70,72]; (**f**) porosity of GTC [46]; (**g**) compressive strength under different curing age [67]; (**h**) bending strength [73]; (**i**) splitting tensile strength [46]; (**j**) correlation between compressive strength and splitting tensile strength [67]; (**k**) compressive strength under different incorporation of steel fiber [71].

Current research on the mechanical properties of GTC is primarily divided into micro-scale and macro-scale investigations, as illustrated in Figure 11a. At the microscopic level, understanding how GT incorporation influences the mechanical performance of concrete helps to clarify the fundamental mechanisms governing strength development. As shown in Figure 11b, during the initial stage of hydration, dicalcium silicate (C_2_S) and tricalcium silicate (C_3_S) form thin films on the surface of cement particles. The electrostatic attraction among ions induces the formation of a water layer surrounding the particles, prompting both cement and silica fume particles to absorb water. As hydration proceeds, C_2_S and C_3_S react to produce calcium silicate hydrate (C-S-H) and calcium hydroxide (Ca(OH)_2_). Due to the pozzolanic activity of graphite tailings, SiO_2_ and Al_2_O_3_ react with Ca(OH)_2_ to form additional cementitious compounds such as C-S-H, thereby enhancing the hydration process of the cementitious matrix. Ultimately, the generated C–S–H gradually fills the pore spaces within the paste, while GT and sand jointly constitute the integrated concrete structure, as shown in Figure 11d.

As shown in Figure 11e, the compressive strength of GTC with 20% GT replacement reached 48.7 MPa at 28 days (w/b = 0.45, cured under standard conditions) [66], representing a 12% increase over the control mix. The overall trend shows that GT incorporation between 10% and 30% yields optimal compressive strength performance. This can be explained from two perspectives: (1) from the perspective of volume filling and pore refinement, the particle size of GT is smaller than that of ordinary river sand, and an appropriate amount of GT can effectively fill fine pores within the concrete, thereby increasing the material’s density. This is substantiated by the measurement of the apparent density of GTC specimens by Jiang et al. (2025) [66], as shown in Table 7, where the concrete with 10% GT incorporation exhibits the highest apparent density, corresponding to the trend in Figure 11e; (2) regarding the hydration reaction, calcium and silicon play key roles in concrete hardening. The incorporation of GT accelerates the hydration process of cementitious materials, forming a denser and stronger cementitious matrix that enhances the internal structure of the concrete. The pore structure can also intuitively reflect the mechanical properties of GTC. Liu et al. (2020) [46] investigated the porosity of GTC, as shown in Figure 11f, and found that when the GT content is below 30%, the internal pore structure remains fine and the matrix is relatively dense.

However, as the GT content continues to increase, the compressive strength of concrete tends to decrease. Excessive addition of GT leads to a reduction in the overall apparent density of the material. Furthermore, the addition of GT absorbs a large amount of free water, resulting in insufficient reaction of cement-based materials, and the decrease in cementitious materials leads to an increase in concrete cracks, weakening its strength and deformation properties [67,68,69]. Nevertheless, Jiang et al. (2025) [66] found that as GT incorporation increases to 40%, the compressive strength of concrete shows a tendency to increase instead. This can be explained from the perspective of a hydration reaction, where the silica-alumina oxides within the graphite tailings exhibit slow activation behavior, generating part of the cementitious material under the stimulation of calcium, thereby increasing the compressive strength of concrete. However, due to the slow reaction, the final increase will not be substantial, as observed more visually in Table 7.

Xue et al. (2019) [67] explored the effect of curing age on the compressive strength of GTC, as shown in Figure 11g. The results indicate that the compressive strength increases with curing age, exhibiting a trend of initial increase, subsequent decrease, and final recovery. Two assumptions are made based on this behavior. First, during the curing process, a portion of the cementitious material may leach out, thereby increasing the internal porosity of the material. Second, the hydration of cementitious materials produces expansive products such as calcite, which cause micro-expansion and additional porosity, leading to a temporary reduction in compressive strength. As curing progresses, the ongoing hydration reactions and the formation of additional cementitious compounds mitigate the increased porosity, resulting in a subsequent increase in the compressive strength of the concrete.

Liu et al. (2020) [70] investigated the effect of GT incorporation on the flexural strength of steel fiber-reinforced concrete, as shown in Figure 10a When the GT content is 10%, the specimens exhibit improved flexural strength. It was found that the inclusion of an appropriate amount of steel fibers can suppress crack propagation, thereby enhancing the flexural performance of the concrete. Tao (2019) [71] also examined the combined influence of GT and steel fibers on the flexural strength of concrete, and their findings indicate that steel fibers have a significant reinforcing effect on GTC. When the steel fiber dosage is 2%, the flexural strength of the concrete increases by 60.62%.

Liu et al. (2020) [46] and Xue et al. (2019) [67] examined the splitting tensile strength of GTC with GT as a sand substitute. As shown in Figure 10b the overall trend indicates that mechanical strengths tend to increase and then decrease with the increase in GT incorporation. This suggests that GT incorporation has a positive effect on improving concrete mechanical properties. Based on this, the relationship between compressive strength and splitting tensile strength of GTC was further investigated [67]. A strong exponential relationship between the two can be observed in Figure 11j, with the coefficient of determination (R^2^) of the fitted equation being as high as 0.9374. The fitted equation is presented in Equation (2).

(2) $${\hat{f}}_{\mathrm{ts}}=0.0033f_{\mathrm{cu}}^{1.9592}$$

where: ${\hat{f}}_{\mathrm{ts}}$ is the theoretical value of flexural strength.

As mentioned in the bending strength section, the incorporation of an appropriate amount of steel fibers in GTC can enhance concrete performance. Through investigations on the effect of steel fibers on GTC, scholars have found that the addition of steel fibers to GT effectively inhibits crack propagation [71]. The steel fibers incorporated into the concrete form a random mesh-like distribution, which impedes the initiation and propagation of microcracks within the concrete during the early stages. Additionally, steel fibers have a high elastic modulus, which helps to bear applied loads and alleviate stress concentrations at crack tips. However, as the steel fiber content increases, achieving a uniform distribution becomes more difficult. As shown in Figure 11k, considering the compressive properties of GTC as an example, the compressive strength of GTC initially increases, then decreases with increasing steel fiber content, with 1.5% being the optimal steel fiber dosage. The improvement of GTC properties by steel fibers is of great research significance.

In summary, the physicochemical properties of GT facilitate concrete hydration; when the GT replacement level is around 20%, the mechanical properties of concrete are optimal. However, as the GT content increases further, the beneficial effect on concrete becomes diminished. Therefore, addressing the adverse impacts of high-GT content on the hydration process and developing high-performance, high-density material systems remain key challenges for future research.

### 5.3. Durability

It should be emphasized that durability indicators discussed in this section mainly include electrical conductivity, shrinkage behavior, chloride resistance, and freeze–thaw resistance. Durability aspects such as carbonation resistance, sulfate attack, and alkali–aggregate reaction have not been systematically investigated for graphite tailings concrete and therefore represent important research gaps.

#### 5.3.1. Conductivity

Graphite tailings contain graphite elements, which, due to the hexagonal planar arrangement of carbon atoms with residual electrons, exhibit relatively high thermal and electrical conductivity compared to conventional concrete. The conductive concrete prepared using GT possesses both the structural characteristics of conventional materials and the electrical conductivity of graphite, making it suitable not only as a structural material but also for applications such as electromagnetic interference shielding, power equipment grounding, cathodic protection of steel reinforcement, and road de-icing and snow melting, among others [74].

Electrical resistivity is an important indicator for evaluating the durability of concrete, and the accurate measurement of GTC resistivity is a topic of considerable research interest. There are generally two methods used to determine the resistivity of GTC: the two-electrode and four-electrode methods. The two-electrode method is widely used in practice due to its simplicity and convenience, whereas the four-electrode method can effectively minimize measurement errors caused by polarization phenomena and provide higher accuracy [75].

**Figure 12:**
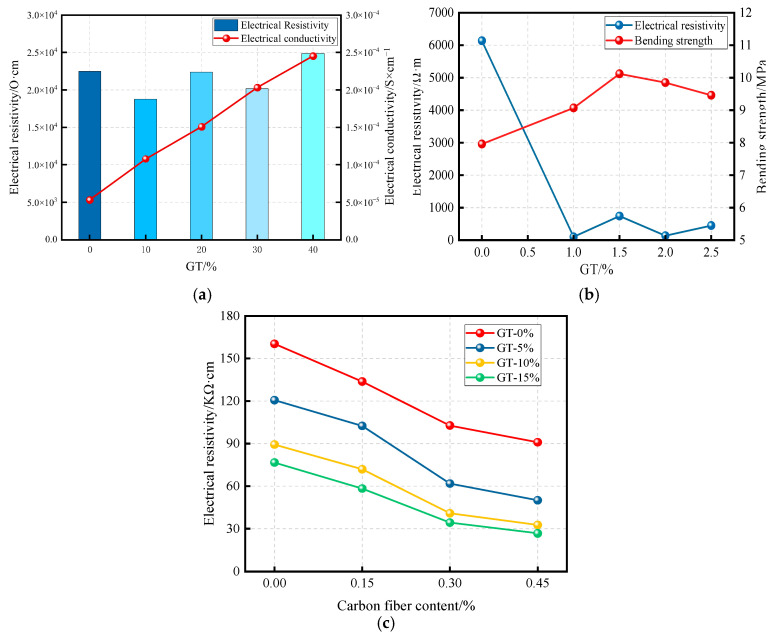
Conductivity of GTC. (**a**) Electrical resistivity and electrical conductivity of GTC [70]; (**b**) electrical resistivity and bending strength of GTC [77]; (**c**) electrical resistivity under the influence of carbon fiber [78].

The resistivity of concrete is influenced by the curing age and generally increases as curing progresses [75]. As shown in Figure 12a,b, GT incorporation affects both the resistivity and electrical conductivity of concrete. However, the variation trends of GT incorporation on compressive strength and resistivity differ significantly. Based on these findings, establishing a correlation between the mechanical properties and resistivity of GTC can provide a theoretical foundation for the future development of green and intelligent concrete structures incorporating GT [70,72,76,77]. Some of the corresponding fitting equations are presented in Table 8.

Traditionally, carbon fibers have been widely used to enhance the electrical conductivity of concrete. However, the high market price of short-cut carbon fibers makes their large-scale application economically prohibitive. In contrast, graphite tailings, as an industrial by-product, offer a low-cost and conductive alternative. Replacing short-cut carbon fibers with GT in concrete can significantly improve the cost-effectiveness of conductive concrete structures. Figure 12 illustrates the electrical resistivity and conductivity of GTC in relation to graphite content, bending strength, and carbon fiber reinforcement. It is observed that the incorporation of both graphite and carbon fibers effectively reduces the electrical resistivity of the concrete. Specifically, when the carbon fiber content is held constant, the resistivity further decreases with the increasing addition of graphite. Similarly, under a fixed GT incorporation, a higher dosage of short-cut carbon fibers also enhances the electrical conductivity of the concrete [78].

#### 5.3.2. Shrinkage and Permeability

**Figure 13:**
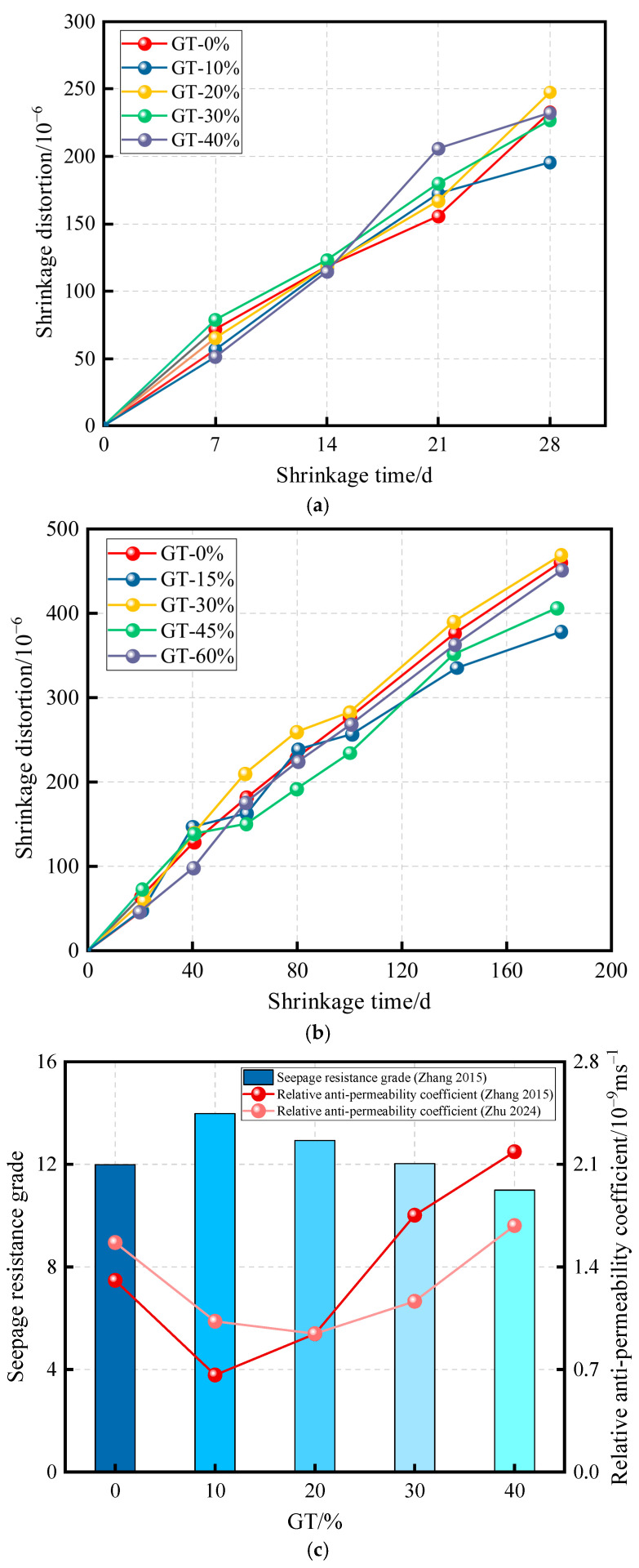
Shrinkage and permeability resistance performance of GTC [81]. (**a**) Early shrinkage deformation; (**b**) late shrinkage deformation; (**c**) permeability resistance performance of GTC, Data sources (from top to bottom): [41,82].

The shrinkage behavior of concrete has always been a critical issue, as it directly affects the overall stability of the structure. For graphite tailings concrete, during early-stage shrinkage deformation, much of the free water within the concrete is adsorbed and retained by GT, leading to an increase in capillary tension and consequently greater shrinkage deformation. As GT incorporation increases, it progressively alters the internal pore structure of the concrete, resulting in different shrinkage behaviors between early and later ages. Figure 13a,b shows the variation in shrinkage deformation of GTC during the early and late stages, respectively. In addition, shrinkage significantly influences the permeability resistance of concrete. Permeability resistance describes the ability of cement-based materials to resist fluid penetration through the pore structure and is commonly assessed by water absorption and permeability-related durability indicators. Low permeability is an important indicator for assessing the durability of concrete. As shown in Figure 13c, a low GT content helps improve the permeability resistance of concrete, with a 10% replacement rate being suitable for practical engineering applications [81].

#### 5.3.3. Resistance to Chloride Attack

**Figure 14:**
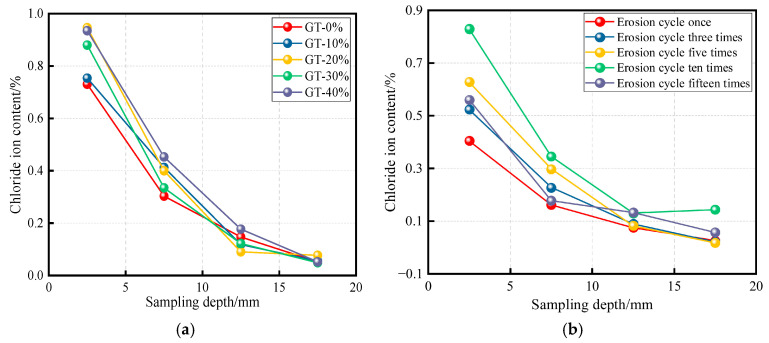
The resistance to chloride ion erosion of GT. (**a**) Effect of GT incorporation [81]; (**b**) effects of erosion cycles [83].

Concrete structures in tidal zones are subjected to cyclic wet–dry conditions, making the study of chloride ion erosion essential for assessing corrosion resistance. Understanding this process is of considerable practical significance. Excessive chloride ion content can compromise structural safety. The free chloride ion content in concrete is evaluated from two perspectives [81]. The first examines the effect of GT incorporation, as shown in Figure 14a. In the near-surface zone, the free chloride ion content in conventional concrete is generally lower than that in GTC. However, with increasing depth, the chloride ion content in GTC decreases more markedly, and the influence of GT incorporation gradually diminishes. The second aspect discusses how the chloride resistance of GTC varies with erosion cycles, as shown in Figure 14b. For instance, at a 15% GT incorporation and fixed sampling depth, the chloride ion content in concrete specimens increases with longer erosion cycles. This is attributed to microstructural changes in the graphite-modified concrete over time. Overall, GT plays a significant role in mitigating chloride ion erosion.

#### 5.3.4. Freeze–Thaw Resistance

**Figure 15:**
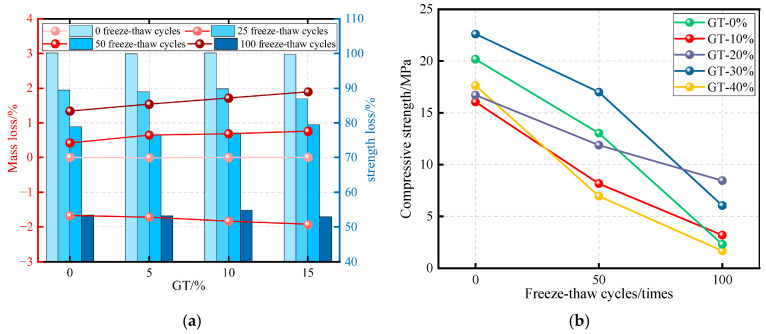
Freeze–thaw resistance of GTC. (**a**) Mass loss and Strength loss of GTC [41]; (**b**) compressive strength of GTC [71].

In the regions where annual temperature fluctuations are significant, concrete structures in freeze–thaw environments undergo repeated freezing and thawing cycles, leading to reductions in both toughness and strength. For GTC, it is essential to evaluate how GT incorporation influences freeze–thaw resistance. Graphite tailings concrete was studied under freeze–thaw conditions to investigate the effect of GT incorporation on the loss of mass and strength of concrete [71]. As shown in Figure 15, the mass and compressive strength of GTC decreased as the number of freeze–thaw cycles increased. GT incorporation was found to have a limited effect on mass loss; however, the compressive strength of concrete decreased with increasing GT content.

### 5.4. Graphite Tailings in Specialty Concrete

#### 5.4.1. Graphite Tailings Foam Concrete

Foam concrete is a lightweight porous material offering advantages over conventional concrete, including superior thermal insulation, seismic resistance, and workability [82]. Sun et al. (2020) [84] investigated the influence of GT incorporation on the strength of foam concrete. As shown in Figure 15, compressive strength reached its peak at 20% GT content, a finding consistent with the results reported by Sun et al. (2021) [85]. The fineness of GT also contributes to strength development, as finer particles disperse more uniformly and integrate more effectively with cement, thereby enhancing mechanical properties. Other researchers have explored high-volume GT incorporation and demonstrated that foam concrete with 60–70% GT still satisfies relevant performance specifications [5,86,87]. These findings suggest promising applications for GT in foam concrete. In one such application, GT-based foam concrete exhibits adequate freeze–thaw resistance, meeting the performance requirements for aircraft runway barrier systems [88,89,90].

#### 5.4.2. Graphite Tailings Autoclaved Aerated Concrete

Wang et al. (2019) [91] investigated the preparation and properties of autoclaved aerated concrete using GT as a siliceous material, combined with cement, quicklime, phosphogypsum, and aluminum powder. Their study confirmed the feasibility of using GT as a siliceous raw material in autoclaved aerated concrete and identified the optimal mix proportions. In a related study, Peng et al. (2021) [92] also utilized GT as a siliceous component and examined the effects of cement content, calcium-to-silica ratio, water-to-solid ratio, and gas-forming agent dosage on the compressive strength and bulk density of the concrete. By optimizing these parameters, they successfully produced graphite tailings-based autoclaved aerated concrete with a compressive strength of 5.0 MPa and a bulk density of 666 kg/m^3^.

### 5.5. Discussions

Durability performance of GT-based cementitious materials shows mixed trends across studies, largely due to non-uniform testing protocols and exposure conditions. Despite these variations, several durability-related mechanisms exhibit relatively consistent behavior. Reduced permeability and improved resistance to sulfate attack are most frequently reported and are commonly attributed to matrix densification induced by fine GT particles. In contrast, findings related to carbonation resistance and freeze–thaw performance remain inconclusive, with both beneficial and detrimental effects reported depending on GT content, curing regime, and pore structure evolution. These discrepancies indicate that certain durability improvements are robust, whereas others are highly sensitive to experimental conditions and require further systematic investigation.

## 6. Graphite Tailings Concrete Structural Components

### 6.1. Graphite Tailings Concrete Beams

Feng et al. (2022) [93] investigated the bending strength of GT-modified concrete beams. Bending tests were conducted on four sets of graphite tailings concrete beam specimens with varying GT substitution rates, lengths of 1600 mm, and cross-sectional dimensions of 120 mm × 180 mm. The test results showed that the failure modes and crack propagation of graphite tailings concrete beams were similar to those of conventional concrete beams. The beams were also found to comply with the plane section assumption, providing a foundation for the theoretical calculation of the ultimate load capacity. Load–deflection curve analysis revealed that the optimal flexural performance was achieved at GT replacement rates between 10% and 20%. However, existing design codes for conventional concrete were not directly applicable for predicting the ultimate bending moment of graphite tailings beams. To address this limitation, a new correction factor was proposed.

**Figure 16:**
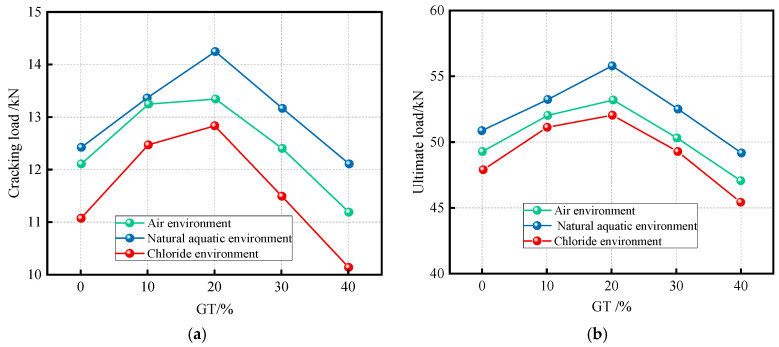
Effect of GT incorporation on cracking load and ultimate load of test beams [94]. (**a**) Cracking load; (**b**) ultimate load.

Feng et al. (2023) [94] studied the flexural capacity of graphite tailings concrete members in chloride environments by conducting bending tests on beams with varying GT replacement levels under three curing conditions: air, water, and chloride salt. As illustrated in Figure 16, the flexural performance initially increases and then decreases with increasing GT content. Notably, the incorporation of GT effectively enhanced both the cracking load and ultimate load of the test beams exposed to chloride conditions.

To more accurately determine the ultimate bearing capacity of graphite tailings concrete beams, Feng et al. (2023) [94] derived a modified calculation formula via Gaussian function fitting, based on test data from chloride-corroded specimens.

(3) $$M^{y}=yM_{u}^{y}$$

(4) $$y=y_{0}+\frac{A}{\omega +\sqrt{\pi /2}}\times \exp{\left[-2\frac{x-\omega_{0}}{\omega }\right]}^{2}$$

where $M^{y}$ is the corrected ultimate bearing capacity of the specimen after chloride corrosion; y is the correction factor; $M_{u}^{y}$ is the theoretical formula for the ultimate bearing capacity of the test beam after chloride corrosion; x is the replacement rate of GT sand; $y_{0}$ = 1.10078; A = 13.81231; ω = 35.40572; $\omega_{0}$ = 17.10824.

### 6.2. Graphite Tailings Concrete Columns

Yang et al. (2021) [95] evaluated the seismic performance of frame columns made with graphene oxide-modified recycled concrete. Their study considered the influence of both graphite content and the oxidation degree of graphene oxide on the properties of the recycled aggregate concrete. By analyzing hysteresis curves, skeleton curves, stiffness degradation, and energy dissipation capacity derived from seismic tests, they demonstrated that the incorporation of graphitic materials can effectively enhance the structural performance of concrete members.

**Figure 17:**
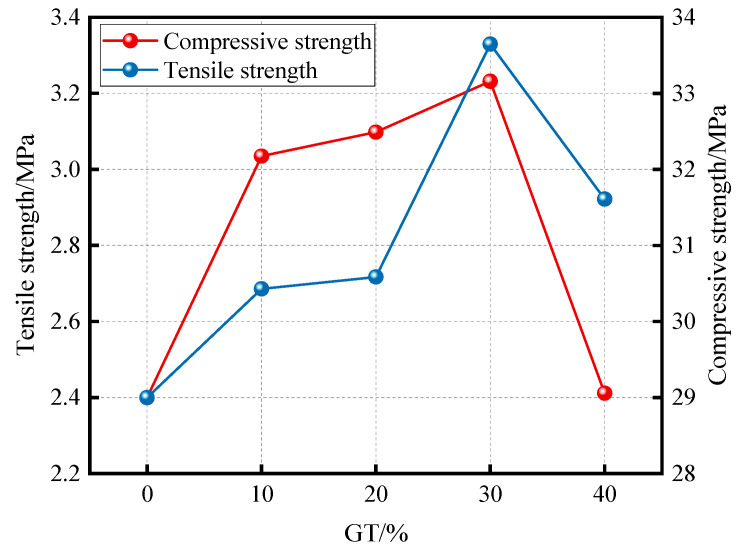
Mechanical properties of graphite tailings concrete columns [96].

Wang et al. (2023) [96] investigated the optimal replacement ratio of graphite tailings (GT) in concrete from the perspective of mechanical performance. Based on the current Chinese specifications, they designed concrete with different GT replacement ratios under the same mixing ratio. Compressive strength, splitting tensile strength, and static elastic modulus were tested. As shown in Figure 17, both tensile and compressive strengths were influenced by GT content, with 30% identified as the optimal replacement ratio. Quasi-static tests were also conducted on both ordinary concrete columns and graphite tailings concrete columns with the optimal replacement ratio, under varying axial compression ratios. Based on the observed failure patterns and hysteresis curves, the hysteretic and skeleton behaviors of graphite tailings concrete columns under low-cycle repeated loading were found to be similar to those of ordinary concrete columns. This indicates that graphite tailings concrete columns can replace conventional concrete columns in seismic applications. Furthermore, the tests revealed that the axial compression ratio significantly affects the seismic failure mode: bending failure dominated at low ratios, while bending-shear failure occurred at high ratios. Therefore, the selection of axial compression ratios should be carefully considered in engineering practice to ensure seismic performance.

### 6.3. Discussions

Existing studies on GTC structural components indicate that, at moderate replacement levels, the incorporation of graphite tailings does not fundamentally alter the structural behavior of reinforced concrete members. Experimental results on beams and columns show failure modes, crack development, and load–deformation responses comparable to those of conventional concrete, supporting the applicability of classical structural assumptions. It should be noted, however, that this conclusion is primarily based on small-scale component tests under monotonic loading, and evidence regarding cyclic, seismic, or system-level behavior remains scarce. The observed improvements in cracking and ultimate capacities are mainly attributed to material-level densification effects, while excessive GT contents may offset these benefits due to cement dilution and increased water demand. However, current research remains limited to a small number of component types and simplified loading conditions, and design methods largely rely on empirical modifications of conventional formulas. Consequently, although the feasibility of GTC in load-bearing structural components has been preliminarily demonstrated, systematic structural-level investigations and rational design models are still required before broader engineering applications can be justified.

## 7. Trends and Future Research Needs

To provide a concise overview of the influence of graphite tailings on cement mortar and concrete, Table 9 summarizes the reported effects of graphite tailings incorporation on the key properties of GTCM and GTC, including mechanical performance and durability-related indicators. Additionally, to facilitate cross-study comparison, the mechanical properties of GT-based cementitious materials reported in the literature are summarized in Table 10 using commonly adopted quantitative indicators, including compressive strength, flexural strength, density, and curing age.

Based on the above analysis, the utilization of GT in construction materials is mainly concentrated in cement mortars and concretes, where substantial research progress has been made. For GTCM and GTC, current studies primarily focus on mechanical behavior and durability performance. In the future, more investigations into the flexural and splitting tensile properties of GT-based building materials are needed to provide a foundation for their practical application in the production of construction materials. Exploring the feasibility of preparing other types of GT-based concretes can also offer a theoretical basis for developing green and intelligent concrete structures. However, GT still exhibit several deficiencies in material properties and construction processes. For example, high fine-particle content and poor interface bonding can reduce strength and ductility, and inadequate control during mixing, casting, or curing may lead to internal defects, compromising both short-term performance and long-term durability. To promote breakthroughs in GT technology, it is essential to increase research investment, introduce advanced techniques, and emphasize independent innovation to enhance material performance and quality.

Research on graphite tailings concrete structural components remains limited, particularly regarding their seismic performance. Current investigations on graphite tailings concrete beams and columns are still in the early exploratory stage and require further improvement in experimental methods. Moreover, studies on other structural elements, such as GTC slabs and joints, are relatively scarce. Therefore, researchers should broaden the domestic research scope as a basis for the in-depth study of GTC structural behavior. On this basis, the overall structural performance of GTC systems should be further analyzed and understood. Such research will also contribute to alleviating environmental problems associated with GT discharge and storage by promoting the maximum utilization of accumulated tailings.

## 8. Conclusions

This paper reviews the characteristics and applications of graphite tailings, analyzes the workability, mechanical properties, and durability of graphite tailings cement mortar and graphite tailings concrete, and summarizes the current state of research on graphite tailings concrete structural components. The following conclusions are drawn:

1. GT possess physicochemical properties (particle size distribution, chemical composition) comparable to natural river sand, fulfilling the fundamental requirements for use as a partial replacement of fine aggregates. Environmental risk assessments indicate that heavy metals (notably Hg) in raw GT require monitoring, but encapsulation in a cementitious matrix significantly reduces leachability. However, the high variability in GT properties, dictated by ore source and processing, poses a key challenge for standardization and predictable performance.
2. At low to moderate replacement levels (10–20% by mass), GT generally enhance the particle packing density, refine the pore structure, and improve the impermeability of both cement mortar (GTCM) and concrete (GTC). This leads to optimal or comparable mechanical properties (compressive, flexural strength) and improved resistance to sulfate attack and chloride ion penetration. A critical limitation is the predominant physical (filler) effect of GT; evidence for significant intrinsic pozzolanic reactivity remains weak and inconsistent across studies, explaining the performance decline at higher replacement rates (>30%) due to cement dilution and increased water demand.
3. The inherent carbon content imparts appreciable electrical conductivity to GTC, enabling non-structural functional applications such as de-icing and sensing. Furthermore, GT have been successfully incorporated into lightweight materials like foamed and autoclaved aerated concrete. The long-term stability of these functional properties and the durability of GT-based lightweight concrete under realistic environmental exposure are major uncertainties requiring validation.
4. Preliminary investigations on beams and columns indicate that GTC with ≤20% GT exhibits structural behavior (failure modes, crack patterns) similar to conventional concrete, supporting the plane-section assumption. Nevertheless, the current experimental evidence is severely limited, presenting the most significant barrier to engineering adoption. The limitations include: (i) a narrow scope of component types (primarily beams under static bending); (ii) a near-total absence of data on seismic, dynamic, and long-term sustained loading performance; (iii) lack of studies integrating material-level durability degradation (e.g., after freeze–thaw cycles) with structural capacity; and (iv) no validated, codifiable design methodologies. Consequently, the promising material-level properties have not yet been translated into reliable structural engineering practice.

In summary, while graphite tailings demonstrate clear potential as a sustainable alternative material in cement-based composites, the existing body of evidence remains insufficient for direct and generalized structural design application, highlighting the need for further systematic investigations, as discussed in the subsequent section.

## 9. Future Trends and Research Needs

Despite the encouraging progress in the utilization of graphite tailings in cement-based materials, significant gaps remain before their reliable application in structural engineering can be achieved. Future studies should move beyond material-level investigations and isolated component tests, and instead focus on the structural performance of graphite tailings concrete members under cyclic, seismic, and other realistic loading conditions. Such studies are essential for evaluating the applicability of existing structural assumptions and for identifying potential GT-related performance limitations.

In addition, greater attention should be given to the coupling between durability degradation and structural capacity. The long-term effects of freeze–thaw cycles, sulfate attack, and chloride-induced deterioration on the mechanical and deformation behavior of graphite tailings concrete remain largely unexplored. Finally, the development of rational, performance-based design methodologies specifically tailored for graphite tailings concrete is required to replace the current reliance on empirical modifications of conventional reinforced concrete design formulas and to facilitate future codification and engineering application.

## Figures and Tables

**Table 1:** GT particle size distribution.

Particle Size/mm	>2.0	2.0~1.0	1.0~0.5	0.5~0.25	0.25~0.1	0.1~0.075	<0.075
Jixi/% [43]	0	0.64	12.30	27.34	38.14	6.99	14.61
Luobei/% [47]	0.30	1.74	79.0	31.3	31.5	10.2	5.78

**Table 2:** Physical properties of GT.

Region	Apparent Density (kg/m^3^)	Bulk Density (kg/m^3^)	Water Absorption (%)	Fineness Modulus	pH	Reference
Jixi Heilongjiang	2855	1540	31.7	0.9	10	[46]
Luobei Heilongjiang	2860	1550	36.9	0.8	--	[48]
Qingdao Shandong	2776	--	--	2.5	--	[49]
Vancouver Canada	--	1700	--	--	--	[50]

**Table 3:** Mineral composition of GT (100% combined) [51].

Mineral	Graphite	Muscovite	Biotite	Quartz	Anorthite	Potash Feldspar
Content/%	61.11	10.36	3.75	7.66	1.27	3.14
Mineral	Albite	Calcite	Hornblende	Pyrite	Apatite	Kaolinite
Content/%	2.31	1.01	2.06	0.06	0.88	0.01
Mineral	Rutile	Sphene	Limonite	Epidote	Tremolite	Other
Content/%	0.01	0.37	0.46	0.50	0.44	4.30

**Table 4:** Chemical composition of GT (99.33% combined) [52].

Chemical Constituents	SiO_2_	Al_2_O_3_	Fe_2_O_3_	MgO	CaO	Na_2_O
Content/%	52.53	11.34	10.38	2.49	10.14	0.76
Chemical constituents	K_2_O	H_2_O	TiO_2_	P_2_O_5_	MnO	L.O.I
Content/%	2.58	0.28	0.49	0.11	0.010	8.50

**Table 5:** Classification standards for heavy metal pollution of soil.

Rating	Single Factor Pollution Index Classification Standard	
	Pollution Index (*P_i_*)	Class of Pollution
First-stage	*P_i_* < 1	Cleaning
Second-stage	1 ≤ *P_i_* < 2	Light pollution
Third-stage	2 ≤ *P_i_* < 3	Moderate pollution
Fourth-stage	*P_i_* ≥ 3	Heavy pollution

**Table 6:** Concentrations of heavy metal elements and contamination indices in graphite tailings fraction.

Element			Zn	Ni	Cr	Hg
Concentration of heavy metals (mg/Kg)	Graphite tailings slag [53]		324.20	128.95	224.82	5.76
	Graphite tailings slag [54]		312.63	132.43	302.25	7.66
	Graphite tailings dam [55]		313.69	101.31	140.58	4.94
	Heilongjiang regional average (Graphite tailings dam) [56]		70.7	22.8	58.3	0.037
	national standard (Graphite tailings dam)	PH > 7.5	≤300	≤60	≤350	≤1
		PH 6.5–7.5	≤250	≤50	≤300	≤0.5
Single factor contaminant index P_i_	Graphite tailings slag [53]		81.0	2.15	0.90	5.76
	Graphite tailings slag [54]		1.04	2.21	0.81	7.66
	(Graphite tailings dam) [55]		3.40	2.93	1.62	16.47
	Heilongjiang regional average (Graphite tailings dam) [56]		4.44	4.44	2.40	133.51
	national standard (Graphite tailings dam)		1.05	1.69	0.40	4.94
Pollution classification	Graphite tailings slag [53]		light	moderate	cleaning	heavy
	Graphite tailings dam [55]		heavy	light	moderate	heavy

**Table 7:** Apparent density of GTC [66].

GT/%	0	10	20	30	40
Apparent density/kg·m^−3^	1947	1992	1928	1891	1940

**Table 8:** Correlation between compressive strength and resistivity of GTC.

Correlation Parameters	Relation Equations	R^2^	Reference
Compressive strength, resistivity	com. = −0.012log_10_(Res.) + 60.022	0.759	[70]
Compressive strength, resistivity	ρ = 0.00014x + 12.88	0.924	[79]
Compressive strength, resistivity, rebound value	f_cu_ = 28.63 − 25.62e^81.41Rm+59.87ρ^	0.979	[80]

**Table 9:** Summary of the properties of GTCM and GTC incorporated with GT.

Property	GTCM	GTC	Remarks
Workability (flow/slump)	Decrease	Decrease	Generally reduced due to finer particle size and higher water demand of GT
Density	Slight increase/No significant change	Slight increase/No significant change	Depends on replacement ratio and GT density
Compressive strength	Increase/Decrease	Increase/Decrease	Strength enhancement at low replacement ratios due to filler effect; reduction at high GT contents
Flexural strength	Increase/No significant change	Mixed	More pronounced improvement reported for mortar than concrete
Splitting tensile strength	Not widely reported	Mixed	Limited studies available; results depend on mix design
Elastic modulus	Slight increase/No significant change	Slight increase/No significant change	Related to matrix densification
Water absorption	Decrease	Decrease	Reduced porosity due to micro-filling effect of fine GT particles
Porosity	Decrease	Decrease	GT improves particle packing and pore structure refinement
Chloride ion penetration resistance	Increase	Increase	Improved impermeability and refined pore structure
Sulfate attack resistance	Increase	Increase	Enhanced resistance attributed to reduced permeability
Freeze–thaw resistance	Not widely reported	Increase/Mixed	Improvement observed at low GT contents; data still limited
Drying shrinkage	Increase	Mixed	Finer particles may increase shrinkage in some cases
Carbonation resistance	Mixed	Mixed	Conflicting results reported in literature
High-temperature resistance	Not reported	Not reported	Insufficient data available
Long-term durability	Increase	Increase	Overall improvement mainly due to densified microstructure

**Table 10:** Properties of GT-based cementitious materials reported in the literature.

Material System	GT Replacement (%)	Curing Age	Compressive Strength (MPa)	Density (kg/m^3^)
GTCM	0	28 d	38–42	2150–2200
GTCM	10	28 d	40–45	2180–2220
GTCM	20	28 d	35–38	2120–2180
GTC	0	28 d	45–50	2350–2400
GTC	15	28 d	47–52	2380–2420

## Data Availability

No new data were created or analyzed in this study. Data sharing is not applicable to this article.
